# Non-alcoholic Steatohepatitis in Asians: Current Perspectives and Future Directions

**DOI:** 10.7759/cureus.42852

**Published:** 2023-08-02

**Authors:** Gourdas Choudhuri, Saumin Shah, Anand Kulkarni, Nitin Jagtap, Pratyusha Gaonkar, Akshay Desai, Charles Adhav

**Affiliations:** 1 Gastroenterology, Fortis Hospital Gurugram, Gurgaon, IND; 2 Gastroenterology, Gujarat Gastro and Vascular Hospital, Surat, IND; 3 Gastroenterology and Hepatology, Asian Institute of Gastroenterology, Hyderabad, IND; 4 Gastroenterology, Asian Institute of Gastroenterology, Hyderabad, IND; 5 Oral Pathology, Pfizer Limited, Mumbai, IND; 6 Pharmacology, Pfizer Limited, Mumbai, IND

**Keywords:** dgat2i/acci, fxr agonist, few ppar-α/δ agonists, thr-ß agonist, fgf analogs, glp-1-gip co-agonist, pharmacotherapy, fibrosis, nash

## Abstract

Non-alcoholic steatohepatitis (NASH) is a subset of non-alcoholic fatty liver disease (NAFLD), which, apart from excess fat in the liver, may be characterised by some level of inflammatory infiltration and fibrogenesis, occasionally progressing to liver cirrhosis or hepatocellular carcinoma (HCC). The objective of the current review is to elucidate the rising prevalence, the role of microbiome and genetics in pathogenesis, diagnostic challenges, and novel treatment alternatives for NASH. Newer diagnostic techniques are being developed since using liver biopsy in a larger population is not a reasonable option and is primarily restricted to clinical research, at least in developing countries. Besides these technical challenges, another important factor leading to deviation from guideline practice is the lack of health insurance coverage in countries like India. It leads to reluctance on the part of physicians and patients to delay required tests to curb out-of-pocket expenditure. There is no cure for NASH, with liver transplantation remaining the last option for those who progress to end-stage liver disease (ESLD) or are detected with early-stage HCC. Thus, lifestyle modification remains the only viable option for many, but compliance and long-term adherence remain major challenges. In obese individuals, bariatric surgery and weight reduction have shown favourable results. In patients with less severe obesity, endoscopic bariatric metabolic therapies (EBMT) are rapidly emerging as less invasive therapies. However, access and acceptability remain poor for these weight reduction methods. Therefore, intense research is being conducted for potential newer drug classes with several agents currently in phase II or III of clinical development. Some of these have demonstrated promising results, such as a reduction in hepatic fat content, and attenuation of fibrosis with an acceptable tolerability profile in phase II studies. The developments in the management of NASH have been fairly encouraging. Further well-designed long-term prospective studies should be undertaken to generate evidence with definitive results.

## Introduction and background

Non-alcoholic fatty liver disease (NAFLD) or more appropriately termed Metabolic (dysfunction) associated fatty liver disease or ‘MAFLD’ is a complex disease modulated by numerous mechanisms including metabolic, genetic, environmental, and gut microbial factors. It has become the most common chronic liver disease (CLD) with a worldwide prevalence of approximately 30% [1-5]. A progressive stage of NAFLD is non-alcoholic steatohepatitis (NASH) which may lead to cirrhosis and hepatocellular carcinoma (HCC) [6,7]. Hepatic steatosis in >5% of hepatocytes in the absence of significant alcohol abuse or any other hepatic disease accompanied by ballooning and inflammation in the liver biopsy confirms NASH [4,5]. It has a greater likelihood of worsening to advanced hepatic fibrosis. NASH is currently the second leading indication for liver transplantation and is projected to become the leading one by the next decade, both globally and in India [8,9]. Besides, it also affects the pathogenesis and prognosis of cardiovascular disease (CVD) and chronic kidney disease (CKD) [10,11]. Owing to its association with extra-hepatic and hepatic diseases, compared to the general population, it has a high all-cause-mortality (25.56 per 1000 person-years) as well as specific hepatic-disease mortality rate (11.77 per 1000 person-years) [12]. However, though NASH is a serious condition associated with severe morbidity and mortality, robust and viable diagnostic tools and treatment for cure are still evolving. The objective of the current review is to elucidate the rising prevalence, the role of microbiome and genetics in pathogenesis, risk factors, diagnostic challenges, and treatment alternatives for NASH. It also delves into emerging therapy targets and drug classes, investigating their efficacy in the treatment of patients with NASH.

## Review

Search methodology

A comprehensive literature review was conducted using Medline and Google Scholar, along with an internet‐based search of publicly available information and peer‐reviewed publications that may not be indexed in the databases mentioned above.

NAFLD and NASH burden: Global and India

Estimating the prevalence of NASH is challenging because none of the general population screening studies have obtained liver histology; consequently, differentiating between NASH and NAFLD (steatosis with or without inflammation) is extremely difficult.

The estimated overall global prevalence of NAFLD in adults is ~32%. Various studies from the USA, Europe, and Asia have reported a prevalence of approximately 22%, 37%, and 31%, respectively in the general population [3]. The prevalence of NAFLD shows wide variation across India ranging from 9% to 53% [13-15]. This wide variation in NAFLD could be attributed to the urban-rural divide as per the Prospective Urban Rural Epidemiology (PURE) cohort study [13,16]. Among patients with NAFLD, 40-68% of patients had progressed to definite biopsy-confirmed NASH and 20% of patients had borderline NASH. In Indian patients, biopsy-proven NASH has been found in >60% of patients and advanced fibrosis (≥F3) in 29-35% of patients [17-19].

NAFLD and NASH exhibit age and gender differences in prevalence. The overall prevalence of NAFLD was significantly higher in men than in women (~39% vs ~25%). Nevertheless, following menopause, this sex difference is reduced or abolished [20]. Among younger patients, both these conditions are 2-3-fold more common in males, but the prevalence of NASH is higher in elderly women [21].

The prevalence of NAFLD and NASH (>65%) is much higher in patients with type 2 diabetes mellitus (T2DM) [22]. People with obesity also have a higher prevalence of NAFLD (90%) and NASH (30%) compared to non-obese (25-30%) [23-25]. Data from various studies in India show a prevalence of up to 77% NAFLD in obese patients in the last decade [13]. NASH was diagnosed in ~60% of those with both diabetes and obesity.

NASH is a causative factor for HCC in almost the same number of patients as viral hepatitis (NASH >17% of patients, hepatitis B (HBV) >20% and hepatitis C (HCV ~17%)) [26]. A systematic analysis of the Global Burden of Disease Study has shown that the age-standardized prevalence of compensated cirrhosis has doubled (33%) and decompensated cirrhosis tripled (55%) due to NASH compared to all other causes [27]. Consequently, NASH-associated HCC and decompensated cirrhosis are the leading indications for liver transplantation in India [13].

Different phases of NAFLD: Progress from healthy to cirrhosis NAFLD

**Figure 1:**
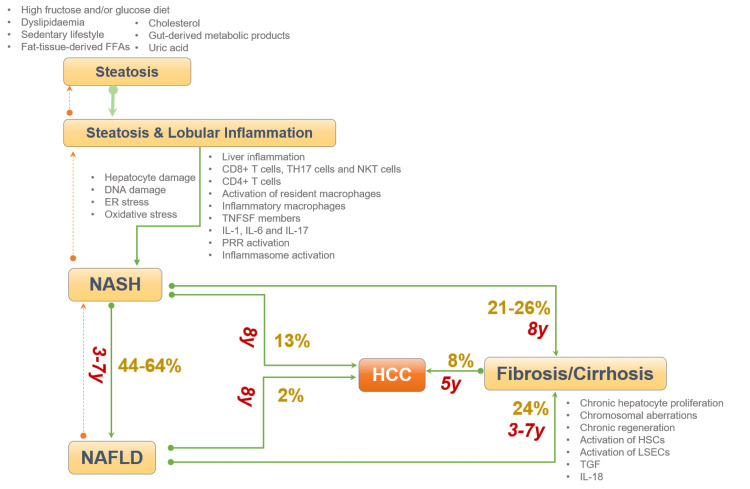
Phases of NAFLD NAFLD-non-alcoholic fatty liver disease, FFA-free fatty acids, IL-interleukin, HSC-hematopoietic stem cell, ER-Endoplasmic reticulum, DNA-deoxyribonucleic acid, HCC-hepatocellular carcinoma, LSEC-liver sinusoidal endothelial cells, NKT-natural killer T-cells, y-years

NAFLD is a complex disease with numerous phases, which involves a diverse degree of intricacies and severity [28]. It begins with simple steatosis progressing to NASH, characterized by inflammatory changes that can lead to progressive hepatic impairment fibrosis and cirrhosis, and could result in HCC (Figure 1) [28].

Role of microbiome in NASH

**Figure 2:**
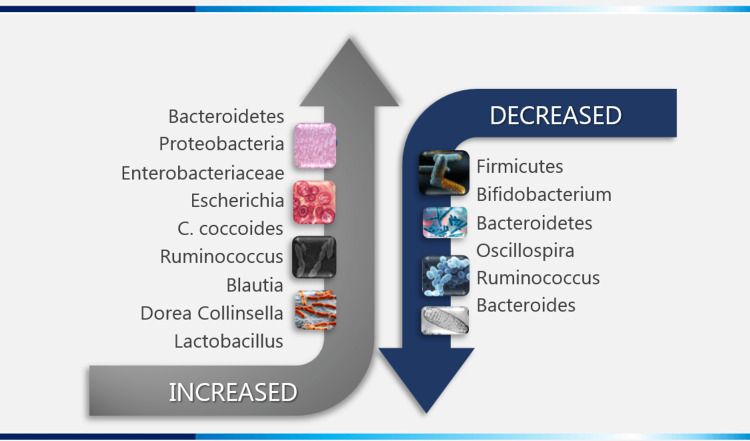
Composition of Intestinal Microbiota in NASH patients NASH-non-alcoholic steatohepatitis, C. coccides-*Clostridium coccoides*

Our gut inhabits 40000 billion microorganisms primarily comprising bacteria, archaea, viruses and fungi, and is denoted as 'microbiota' [29-31]. Previously it has been suggested that these microorganisms are linked with all metabolic diseases including NASH [32]. Though inconsistently reported, dysbiosis with large heterogeneity in microbiota composition exists in patients with NASH and may be associated with its inception and progression (Figure 2) [33]. Studies in patients with newly diagnosed and long-standing T2D showed trans-domain dysbiosis due to interlinked metabolic interactions among the microbiota [31]. Studies have also shown the prominence of Bacteroides and a remarkably high archaeal density with elevated faecal short-chain fatty acid (SCFA) levels in obese subjects [34].

Mechanisms by which the gut microbiota impact the progression to NASH are being explored and a few have been described using experimental data (Table 1) [35].

**Table 1:** Role of the Microbiota in NASH NASH-non-alcoholic steatohepatitis, AMP-antimicrobial peptide, damage-associated molecular patterns, FXR-Farnesoid-X Receptor, GLP-1-glucagon-like peptide 1, IR-insulin resistance, LPS-lipopolysaccharide, NF-KB-nuclear factor kappa-B, NLR-nod-like receptors, PAMPS-pathogen‐associated molecular patterns, SCFA-short-chain fatty acids; TG-triglyceride; TLR-toll-like receptors, Treg-regulatory T cells; TMAO-trimethylamine N-oxide

Parameters	Effect of Dysbiosis on Gut and Liver	Effect on Pathogenesis of NASH
SCFA	1. Decrease SCFA production, 2. Disturb the integrity of the intestinal barrier	Loss of intestinal wall integrity and dysregulated hepatic metabolism Increased hepatic steatosis, inflammation
FXR	Insufficient activation of FXR thus reduced activity	Encourages hepatic steatosis and insulin resistance, as well as negative feedback, inhibits bile acid synthesis
TGR5	Insufficient activation of TGR5 thus reduced activity	Increases inflammation and IR
Trimethylamine	1. Decreases choline levels and increases toxic choline metabolites. 2. Suppresses the activation of liver FXR signaling. 3.Upregulates glucose metabolism and increases IR. 4.Induce the activation of the NF-kB pathway, promote oxidative stress and activate the NLRP3 inflammasome. 5. Increases the release of inflammatory cytokines (IL-18 and IL-1b).	Effect of TMAO on NASH is controversial
LPS	1. LPS deteriorates NASH progression. 2. Induces hepatic inflammatory response and fibrosis via LPS/TLR4 and NFkB signaling pathways in hepatocytes, HSCs, and Kupfer cells. 3. Activates CD14-TLR4 promoting the release of inflammatory cytokines (NF-kB). 4. Induces the activation of macrophages and platelets through the TLR4 pathway, thereby eliciting liver damage. 5. Promotes the expression of TGF-β, which induces the transcription of certain pathways promoting hepatic fibrosis. 6. Induces oxidative stress.	Hepatic inflammation, fibrosis, and liver injury
Activation of Inflammasomes NLRP3	Activates NLRP3 by promoting the entry of PAMPs, DAMPs, and LPS into the portal circulation through the impaired intestinal barrier, resulting in liver inflammation. Instigates IR.	Participates in the transition from NAFLD to NASH to hepatic fibrosis through the TLR4-NF-kB signalling pathway
NLRP6	Deletion of NLRP6 alters the configuration of the intestinal microbiota, resulting in hepatic steatosis and inflammation via TLR4 signaling.	Increased hepatic steatosis
Intestinal dysbiosis	1. Increases abundance of Escherichia. 2. Increases the production of endogenous alcohol. 3. Increases the expression of intestinal inflammatory factors and destroys the intestinal barrier, associated with small intestinal bacterial overgrowth, and aggravates intestinal dysbiosis. 4. Endogenous alcohol inhibits the TCA cycle and aggravates hepatic TG accumulation and deposition. 5. Toxic intermediates of alcohol metabolism (acetaldehyde) impair the function of intestinal tight junction proteins.	Aggravation of hepatic steatosis, inflammation, mitochondrial dysfunction, and liver injury promotes the progress of NASH.

Currently, however, these compositional changes and mechanisms of pathogenesis of microbiota with NASH are vague and inconsistent. Nevertheless, due to a lack of approved alternatives for the prevention of NASH, microbiota-modifying agents such as probiotics, prebiotics, synbiotics, and faecal microbiota transplantation (FMT) are being explored [36-47]. These agents alter intestinal permeability, assuage oxidative stress, and endotoxin release, reduce serum alanine aminotransferase (ALT), aspartate aminotransferase (AST) levels, hepatic inflammation, increase the copiousness of *Faecal bacterium prausnitzii* and *Bifidobacterium *all of which were beneficial in NASH therapy. Besides synbiotics inhibit NASH with improvement in hepatic histology and attenuation of subclinical inflammation. These actions effectively reduce hepatic steatosis and improve BMI and waist circumference (WC). Experimental studies with FMT have shown some benefits, however, its development is still in the infancy for NASH.

Role of genetics

NASH is related to gene-environment interactions. Some patients with NASH are known as rapid progressors which could be due to altered metabolic milieu and increased injury to the liver, or genetic predisposition to scar more destructively. Studies have shown that hepatic steatosis and fibrosis in families occur with a heritability value of ~ 0.5 in age-, gender- and ethnicity-adjusted analysis [48]. The risk of advanced fibrosis was noted to be 12-fold higher in first-degree relatives of people with NAFLD and cirrhosis than in the general population [49]. Genome-wide association studies and candidate gene studies have deciphered variations in genes increasing the susceptibility of individuals to the development of NASH namely patatin-like phospholipase domain-containing protein 3 (PNPLA3), transmembrane 6 superfamily member 2 (TM6SF2), membrane-bound O-acyltransferase domain-containing 7 genes (MBOAT7), glucokinase regulator (GCKR), hydroxysteroid 17-beta dehydrogenase-13 (HSD17B13) [50,51]. The majority of data has been established for the PNPLA3 gene found in 15% of the general population, in which a mutation at position 148 is linked to 2.5-fold greater odds of the development of severe steatohepatitis, excessive, inflammation, injury and scarring; consequently, bearing higher risks of NASH, progression to cirrhosis and HCC [52]. Similar to PNPLA3 are TM6SF2, GCKR and MBOAT7 mutations which increase the odds of promoting NASH progression by 1.18 to 1.55 fold but are rarer compared to PNPLA3 [53-55]. Another variant is HSD17B13 which is rare, however, it is a protective variant that neutralizes some of the deleterious effects of the PNPLA3 gene in people with both gene variants. The risk of NASH was reduced by 14% in the presence of the HSD17B13 variant [56]. Thus, the impact of these mutations on NASH depends on their numbers in a particular individual. A polygenic score helps to quantitatively determine the composite risk of disease progression based on the proportion of deleterious and protective genes. Though the evidence is currently sparse, it has been suggested that genetic factors also determine the response to NASH therapy e.g., decreased effect of polyunsaturated fatty acids (PUFA) or fish oil in patients with fatty liver with PNPLA3 variant [57].

Lean NASH

First reported in Asia, lean NASH is now recognized globally [58]. It refers to individuals with normal BMI (25 kg/m^2^ in Caucasians and 23 kg/m^2 ^in Asian subjects) manifesting the disease. They have excess visceral adiposity and insulin resistance, as well as a metabolic dysfunction, aptly referred to as metabolically obese normal-weight (MONW individuals). Though Lean NAFLD is similar, not identical to NAFLD, accumulating evidence argues that lean NAFLD might be a distinct pathophysiological outlier, with more than half (47-65%) having NASH [58].

Incidence

The MONW individuals are seemingly distributed along racial and ethnic lines, with Asians developing significant metabolic disease outcomes at lower BMIs than other ethnic groups. The prevalence of lean NAFLD has been described in different ethnic populations, mainly Asian (Table 2) [59-66]. These patients too exhibit the whole spectrum of the histopathological characteristics of NASH.

**Table 2:** Prevalence of Lean NAFLD in the Asian Population NAFLD-non-alcoholic fatty liver disease, BMI-body mass index, CT-computed tomography, HDL-high-density lipoprotein, HOMA-IR-homeostatic model assessment-insulin resistance, PNPLA3 G-patatin-like phospholipase domain-containing protein 3 gene, TG-triglycerides, WC-waist circumference

Country	BMI kg/m^2^	N	NAFLD (%)	Risk Factor
China [59]	<25	6905	7.27%	Age, gender, BMI, WC, TG, HDL cholesterol, serum uric acid, hemoglobin, and platelet count
China [60]	<24	2000	18%	Metabolic syndrome
Korea [61]	<25	3014	12.6%	Central adiposity
Korea [62]	<25	1487	22.4%	Male gender, WC, TG, IR
Japan [63]	<25	3271	15.2% vs 68.5% in obese	WC, body fat percent
Hongkong [64]	<23	911	19.3% vs 61 % in obese	Metabolic syndrome and PNPLA3 G allele
India [65]	<25	1911	8.6%	Higher bicep skin fold thickness
India [66]	<23	150	15.3%	IR

The lean individuals with NAFLD were phenotypically distinct: excess subcutaneous fat elevated fasting plasma glucose (FPG) and triglycerides (TG) [63-65]. Metabolic factors such as WC and TGs are predictors of lean NAFLD. Apart from these, weight gain in early adulthood is significantly associated with NAFLD in lean subjects [63]. Lean NASH is similar to obese NASH from a biological perspective; nevertheless, the lack of comparatively less adiposity implies the existence of plausible genetic risk [67].

Outcomes in LEAN NAFLD/NASH

Considering the phenotype of lean NASH within the context of hepatic steatosis, a study in Indian subjects (BMI <23 kg/m^2^) found the prevalence of biopsy-proven NAFLD to be ~8% [65]. Among these patients with NAFLD, 31% had NASH and >2% had cirrhosis. The largest and longest series of patients with biopsy-proven NAFLD with a mean follow-up time of >19 years found a high rate (19%) of NAFLD in lean subjects [68]. Though there was no increased mortality in lean compared to overweight subjects, they were at higher risk, for the development of severe liver disease (decompensated liver disease, liver failure, HCC, or cirrhosis) [68]. Those who developed the severe liver disease in the lean NAFLD group were older, had more severe portal inflammation, fibrosis stage 3 or 4, and a higher prevalence of NASH compared to those who did not [69]. Half of the lean patients with NAFLD had biopsy-proven NASH, which is much higher than that reported 30% in general population studies [70].

Another study in the US cohort which made a similar comparison found that lean patients developed significantly more severe lobular inflammation than the non-lean NAFLD group [71]. Though there were no differences in the proportion of patients with NASH or hepatocyte ballooning, the age-adjusted cumulative survival was significantly shorter in patients with lean NAFLD as compared to those with non-lean NAFLD [71].

Contrary to the findings from Indian and US-based studies, a recently published study with a predominantly European population showed that lean patients had significantly less severe histological disease and less advanced fibrosis. There were no significant differences in the prevalence of the PNPLA3 variant. Further follow-up for ~8 years found no significant difference in hepatic events or survival [72].

A retrospective study analyzed data from the United Network for Organ Sharing (UNOS) database. A comparison of survival after liver transplant in lean and obese individuals after adjusting for confounders found that all obesity cohorts with NASH had significantly reduced risk of graft and patient loss at 10 years of follow-up compared with the lean BMI cohort [73].

Thus, clinical evidence regarding survival and histological disease severity is conflicting across races in lean and non-lean patients with NASH, and more evidence is needed before drawing definite conclusions. Nevertheless, these data do highlight the implication of a genetic link in the absence of excess adiposity in NAFLD/NASH pathogenesis and serious negative clinical outcomes.

Risk factors for NASH

The susceptibility and progression of NASH are ascribed to the dynamic interaction between environmental and genetic risk factors. Obesity, older age, female sex, non-African American race/ethnicity, diabetes mellitus, and hypertension are some of the risk factors that increase the probability of NASH (Table 3). Metabolic inflexibility or the inability of the body to maintain balance or to adequately treat/regulate substrates at necessary times is a major contributor. The liver is incapable of shifting back and forth between prandial and fasting states compliantly because of exacerbated insulin resistance which is an indication of NAFLD/NASH. Metabolic inflexibility is associated with hyperinsulinemia and systemic lipotoxic cell stress resulting in inflammation and fibrogenesis, and ultimately NASH [74]. Metabolic inflexibility is higher in people with obesity and T2DM.

**Table 3:** Risk Factors for NASH ADIPOQ gene-Adiponectin, C1Q and Collagen Domain Containing, AHR-aryl hydrocarbon receptor, CIH-Chronic Intermittent Hypoxia, FADS1-fatty acid desaturase 1, FFA-free fatty acid, MRS-magnetic resonance spectroscopy, NAFLD-nonalcoholic fatty liver disease, NASH-non-alcoholic steatohepatitis; OSA-Obstructive Sleep Apnoea, PCOS-polycystic ovarian syndrome, LEP gene-leptin gene, TGF-B-transforming growth factor beta*rate-limiting enzymes for PUFA conversion and are recognized as main determinants of PUFA levels

Risk factor	Mechanism of Interactions	Illustrative Evidence
Diet and extrahepatic milieu	High‐calorie diets, excessive consumption of sugar promotes obesity and excess adipose tissue leading to excess inflammatory cytokine formation Affects the intestinal microbiome and alters intestinal permeability, exposure to endotoxins sets an inflammatory cascade. Provides excessive FFA to the liver both directly and by promoting IR Metabolic inflexibility, energy imbalance scale causing lipotoxic cell stress	Subjects with NAFLD had a 2 to a 3-fold higher intake of fructose from sugary sweetened beverages than healthy controls [75,76]. Fructose consumption increases in liver fat confirmed by MRS [77]. Its restriction reduces liver fat and de novo lipogenesis in subjects with high baseline fructose consumers compared to an isocaloric diet [78].
Central Adiposity	Visceral fat is a predictor of hepatic steatosis, hyperinsulinemia, decreased hepatic insulin extraction, and peripheral IR. Lipolysis in VAT is more resistant to insulin providing hepatoxic FAs in hyperinsulinaemic states.	Decreasing visceral fat has also been shown to decrease hepatic IR. Lean NASH patients may have central adiposity.
Metabolic syndrome	Fatty liver is the hepatic component of the IR syndrome (IRS)	The risk of hepatic steatosis increased exponentially with each addition of the components of the IRS The presence of the metabolic syndrome makes it more likely that a patient will have NASH rather than steatosis
Obesity	Lipid-laden hepatocytes act as a reservoir for hepatotoxic agents that are more vulnerable to successive injury by endotoxins and cytokines in the production of ROS which causes lipid peroxidation and activation of cytokines, stimulating fibrogenesis and causing cell death Adipocytes are endocrine tissue secreting TNF, resistin, leptin, and free fatty acids that may induce/enhance IR A decrease in adiponectin decreased insulin sensitivity	Though NASH can occur in lean subjects, 57-93% of patients are overweight or obese [79].
Diabetes (Bidirectional)	IR, oxidative stress, metabolic inflexibility	Prevalence of NASH among the diabetic population was 37.3% compared to 3-5% in the general population and a significantly high proportion of developing advanced NASH fibrosis (17%)[80]
Hypertension (Bidirectional)	Inflammation, renin-angiotensin system-sympathetic nervous system activation and insulin resistance, endothelial dysfunction Shared genes among hypertension, NAFLD, fibrosis, and inflammation include LEP, ADIPOQ, AHR and TGFB1 [81].	Epidemiological evidence shows 49.5% NAFLD prevalence in hypertension patients, which is higher than in the general population [81] 1.63 times greater risk of NAFLD [82] Prevalence is 16.5%, 37.5%, and 59.3% in normotensive, pre-hypertensive and hypertensive population [83]
Polycystic ovarian syndrome (PCOS)	The aberrant metabolic and hormonal milieu [84]	Women with vs without PCOS 1. Higher proportion had severe ballooning (32 vs 13%, p=0.02), 2. Presence of any fibrosis (84 vs 66%, p=0.06), 3. Presence of advanced fibrosis (16 vs 6%, p=0.10). 4. Age- and BMI-adjusted analysis found >3-fold greater risk of severe hepatocyte ballooning (p=0.03) and 7-fold greater risk of advanced fibrosis (p=0.02). 5. The median age was 5 years younger in advanced fibrosis (40 vs 45 years, p=0.02)
Obstructive sleep apnea (OSA)	May facilitate the progression of hepatic steatosis to NASH. Induced CIH resembling OSA causes hepatocyte injury, increases lipolysis, and oxidative stress, and up-regulates hypoxia-inducible factor 1, which may increase hepatic steatosis, induce necro-inflammation and fibrogenesis [85]	Three-fold greater risk of NASH in patients with OSA than without OSA after adjusting for confounders (2% vs 0.65%; p<0.0001)
Sarcopenia Muscle and adipose tissue	Muscle- key metabolic organ and buffers the functions of the liver Disrupted adipose–liver–muscle axis in NAFLD due to dysfunctional myokines [74] Ectopic fat storage in muscles causing IR	Studies in Asian populations showed that sarcopenia is associated with the presence and severity of NAFLD [86] Prevalence of sarcopenia in subjects without NAFLD, with NAFL, and with NASH were 8.7%, 17.9%, and 35.0% [86] Skeletal muscle steatosis increased significantly with increasing stage of NASH [87]
Metabolic stress on Liver			Lipoapoptosis is a principal feature of NASH that results from the failure of hepatocytes to dispose of excess FFAs [74].	Biopsies of patients with NASH show decreased activity of FADS1 genes (encoding Liver Delta-6D and Delta-5D activities)* which is a key player in accumulating toxic lipids during NASH progression [88,89]

Diagnostic challenges and unmet needs of NASH

Differentiating NASH from simple steatosis has significant prognostic consequences. However, conventional means of determining plasma liver aminotransferase levels can be unreliable and normal (<40IU/L) in many cases of NAFLD and even in those with clinically significant fibrosis. Given the serious morbidities Figure 1 and the growing need for liver transplantation, it is critical to accurately recognize and diagnose NASH in those with NAFLD and its risk factors [6-9]. Imaging techniques and biomarker panels have been evolving for the diagnosis of NASH/NAFLD. The features and challenges of these methods are depicted in Tables 4-6 [90-92].

**Table 4:** Features and Challenges with Current Imaging Modalities ARFI-Acoustic radiation force impulse, CT-Computed tomography, MRE-Magnetic resonance elastography, MRI-Magnetic resonance imaging, SWV-Shear wave velocity, TE-Transient elastography, US-Ultrasound, VCTE-vibration-controlled transient elastography

Modality	Features	Challenges
US	Widespread availability Inexpensive compared to other techniques	Cannot differentiate necro-inflammation and fibrosis from simple steatosis, so cannot be used for the evaluation of steatohepatitis Limited use in obese and pre-existing liver diseases Low sensitivity if liver fat is <30% Subjective interpretation-highly operator dependent Not routinely recommended
CT	Widespread availability	Cannot differentiate necro-inflammation and fibrosis from simple steatosis, so cannot be used for the evaluation of steatohepatitis Potential radiation exposure Expensive
MRI	Evaluation of the whole liver Accurate for steatosis	Cannot differentiate necro-inflammation and fibrosis from simple steatosis, so cannot be used for the evaluation of steatohepatitis Expensive Susceptible to biases (T1 bias, T2 decay)
TE-VCTE	Good predictive performance to predict cirrhosis in lean patients Can reliably differentiate various stages of fibrosis Useful and cost-effective in NASH Can be performed at the point of care Takes minutes to perform the test- results are instantaneous No sedation is required Preferred over the US as it can quantify liver fat and fibrosis for risk stratification during the same testing	No precise cut-off levels for different stages of fibrosis Difficult to perform in obese patients due to reduction in transmitted vibrations by fatty tissue Region of interest is smaller compared to MRE (1 cm X 4 cm vs entire liver 10.5 cm X 16 cm)
ARFI	Similar diagnostic efficacy with TE Provides both a qualitative measure of displacement and a quantitative measure of SWV	Difficult to perform in obese patients Measurements can be complicated by steatosis and hepatic inflammation
MRE	Highly accurate for detecting liver fibrosis because it can accurately quantify lipid fraction relative to water in tissue and can be used for the assessment of fat content Can analyse the entire liver	Expensive Limited availability Does not replace the gold-standard ‘biopsy’ for NASH diagnosis

**Table 5:** Features and Limitations of Biochemical Markers AUROC-area under receiver operating characteristic, CLD-chronic liver disease, NASH-non-alcoholic steatohepatitis, Fuc-Hpt-Fucosylated haptoglobin, HA-hyaluronic acid

Marker	Mechanism	Features	Limitations
HA	Serum levels are dependent on production, increase with increased collagen synthesis, as well as degradation, which occurs in liver sinusoidal endothelial cells	Correlates well with hepatic fibrosis AUROC: 0.89 for advanced fibrosis	Levels change according to the fasting status of patients Displays large intraindividual variations in both health and CLD
Fuc-Hpt	Glycoprotein is secreted into bile, but not into sera in the normal liver while these increase NASH (ballooning hepatocytes)	Distinguish NASH from simple steatosis AUROC: 0.73 of biopsy-proven NASH 0.72 for detection of advanced fibrosis	-
Mac-2 binding protein	Undetectable in normal liver but is easily detected in NASH patients due to a significant increase in It closely correlates with the fibrosis severity and hepatocyte ballooning	AUROC 0.81 for NASH	-
Fuc-Hpt-Mac-2bp combination		AUROC: 0.85	-

**Table 6:** Features and Limitations of Biomarker Panels AAR-AST-ALT ratio, AST-Aspartate transaminase, ALT-Alanine transaminase, AUROC-area under the receiver operating characteristics, α2-MG-Alpha 2 macroglobulin, ALP-A1- Apolipoprotein A1, BMI-Body mass index, DM-Diabetes Mellitus, ELF-enhanced liver fibrosis test, GGT-Gamma glutamyl-transferase, HA-Hyaluronic acid, HOMA-Homeostatic model assessment, IRI-Immunoreactive insulin, IFG-impaired fasting glucose, NASH-non-alcoholic steatohepatitis, P3NP-Aminoterminal peptide of pro-collagen 3, T2DM-Type 2 Diabetes Mellitus, TIMP1-Tissue inhibitor matrix metalloproteinase 1

Modality	Combination	Utility	Limitations
FibroTest	Age, gender, bilirubin, GGT, ALP-A1, haptoglobulin, α2-MG	AUROC: 0.84 for advanced fibrosis	Cannot show different degrees of fibrosis Failure in Gilbert syndrome, cholestasis, and acute inflammation For distinguishing minimal fibrosis from intermediate fibrosis, it falls short (AUROC for F1 vs F0: 0.53) and a liver biopsy is still needed for definitive staging
ELF	TIMP1, HA, P3NP, BMI, DM/IFG, AST/ALT, platelets, albumin	Has a high sensitivity & specificity May show different stages of fibrosis Can predict liver-related clinical outcomes AUROC: 0.98 for advanced, 0.93 for moderate fibrosis	-
Hepascore	HA, α2-MG, bilirubin, GGT, age, gender	Highly accurate in detecting advanced fibrosis AUROC: 0.90 for cirrhosis	Elevated liver enzymes with unknown etiology, reduce the predictive ability
NAFLD Fibrosis Score	Age, BMI, IFG/T2DM, AST, ALT, platelet, albumin	Most extensively validated system May show different stages of fibrosis AUROC: 0.88 for advanced fibrosis Can avoid liver biopsy in 3 of 4 NAFLD patients with suspected fibrosis	Scores between two cut-off values are common
Fibrosis-4 (FIB-4)	Age, ALT, AST, platelets	Easy, simple, and inexpensive with quick results AUROC: 0.85-0.87 for advanced fibrosis More useful in moderate to advanced fibrosis cases where it can reduce the number of liver biopsies	False-negative results Young age and normal platelet count may cause FIB-4 failure Cannot distinguish between simple steatosis and NASH
Fatty liver Index	BMI, waist circumference, triglyceride, GGT	Correlates well with US images in steatosis AUROC: 0.84 for detecting fatty liver	Cannot show the presence of NASH or fibrosis
Index of NASH	Waist-to-hip ratio, triglyceride, ALT, HOMA	AUROC: 0.88 for steatohepatitis A score of ≥50 has 92% specificity for NASH	Needs to be externally validated
BARD	BMI, AST, ALT, T2DM	Easy to measure AUROC: 0.80 for advanced fibrosis (0.88 with the addition of INR to measurement)	Sensitivity and specificity are lower than in other panels High false positivity
AAR	AST, ALT	Easy to measure	High false positivity in alcohol users
APRI	AST, platelets	Easy and cheap to measure Can exclude significant fibrosis AUROC: 0.76 for advanced fibrosis	Cannot show different degrees of fibrosis

Clinical biomarker panels combine routinely assessed clinical variables like age and the presence of diabetes with different biochemical parameters such as routine biochemical tests, markers of hepatocyte apoptosis, hepatic collagen matrix remodelling and/or adipose tissue-released cytokines (Table 6). Panels for the diagnosis of fibrosis have good specificity and negative predictive value (NPV) that allow the clinician to rule out advanced fibrosis, but they lack adequate sensitivity and positive predictive value (PPV) to establish the presence of advanced fibrosis. Therefore, several individuals fall in the “indeterminate-risk” group and need to be further evaluated with an ELF test. The performance of the panel depends on the population being studied, with a better performance in people with advanced diseases [92].

Liver biopsy remains the “gold standard” for the diagnosis of NASH. Traditionally it is performed via a percutaneous route under ultrasound guidance. Endoscopic ultrasound (EUS) guided liver biopsy is an emerging procedure that offers an alternative to conventional percutaneous and transjugular liver biopsy. The safety and efficacy of EUS liver biopsy and confirming histopathological diagnosis have been evaluated in various studies [93,94]. Biopsies via endoscopic ultrasound or transjugular biopsies by interventional radiology have the additional benefit of measuring the portosystemic pressure gradient which can potentially have an implication on management. However, biopsy by any method should not be used as a screening method to diagnose NAFLD given its multiple limitations. It is invasive and associated with potential adverse effects, such as pain, bleeding and infection, and has poor acceptability. It requires expertise and suffers intra-observer and interobserver variability and sampling variability. Considering these aspects and the patient perspective that it least impacts the choice of treatment alternatives in clinical practice settings, biopsies either by the traditional or endoscopic method are impractical to use in large populations [92]. It is primarily limited to research for the assessment of clinical endpoints. Alternatively, though not validated, non-invasive tests both blood tests and imaging studies are used for the diagnosis of NASH and serve the purpose for choice of appropriate management [92,95].

Another important factor leading to deviation from guideline practice is the lack of health insurance coverage. Lack of health insurance coverage and inadequate coverage are important reasons for high out-of-pocket health expenditures. It typically forces people to delay or postpone medical care. The doctor’s reluctance to advise the patients to undergo various diagnostic tests due to out-of-pocket expenditure. Thus, resource constraints result in the avoidance of recommended tests and the frequency of follow-up tests to control the expenditure. This is specifically an important factor in countries like India where out-of-pocket expenses account for about 62.6% of total health expenditure - one of the highest in the world as per a recent study [96].

Non-pharmacological, pharmacological and surgical alternatives for the treatment of NASH

Interventions for Weight Loss

Diet and lifestyle modifications: Weight loss is a basis of management of NASH patients, improving histology along with the cardio-metabolic profile and glucose homeostasis [97,98]. Clinical evidence suggests that 5-10% weight loss results in an improvement in steatohepatitis in 58-90% of patients with NASH [99]. Similar improvement (40%) has also been reported in the Asian patient population with 3-5% weight reduction [100,101]. In obese patients with NASH ≥ 10% weight loss has also shown significant regression of hepatic fibrosis [102]. Not only obese but lean patients with NASH can also benefit from dietary modification as well as weight reduction strategies [103]. A 12-month study comparing lean and non-lean patients found that any amount of weight reduction was associated with significantly improved steatosis, ballooning and NAS scores in both groups [104]. These data support the notion that weight loss in terms of loss of adiposity improves histology in patients with NASH regardless of baseline BMI [105]. Weight reduction should be achieved by caloric restriction (CR) or implementing low-carbohydrate, low-fat, and Mediterranean-type diets.

Exercise: Exercise has demonstrated benefits in terms of weight loss in patients with NASH with its duration being proportional to the improvement of hepatic steatosis [97,106]. Evidence demonstrating the advantage of one exercise type (aerobic, resistance, high-intensity, or low-intensity exercise) over another is inconsistent [97,107]. Resistance exercises may be more practicable in NASH patients unable to follow aerobic exercise, while in those with limited time availability high-intensity exercise programs may help. The recommended frequency for exercise is more than 30 minutes a day for five days a week [108]. A five-year study demonstrated that high-intensity and moderate exercise were similarly effective in the reduction of intrahepatic TG, reduction in the risk of development of new fatty liver or the resolution of pre-existing fatty liver, primarily through weight loss [109,110].

Combination of diet and exercise: A comparison of CR and aerobic exercise with CR alone in a short-term study found significant improvement in blood pressure, FPG, TG, homeostasis model assessment-estimated insulin resistance, US grading of steatosis, and quality of life only in patients with NASH who followed aerobic exercise [111]. Another long-term study in patients with biopsy-proven NASH following CR and exercise intervention with weight loss ≥ 7% achieved a greater reduction in NAS (-3.45 vs -1.18, P < 0.001), steatosis (-1.36 vs -0.41, P < 0.001) and lobular inflammation (0.82 vs -0.24, P = 0.03) compared to those with <7% weight loss [112]. Thus, there is ample evidence from short- and long-term studies showing partial or complete resolution of NASH or improvement in histology or regression of fibrosis with lifestyle interventions [113,114]. Maintenance of the weight loss is indeed challenging due to its association with changes in dietary habits, and lifestyle. This necessitates a diet plan and exercise type based on patients’ preferences to ensure long-term adherence.

**Figure 3:**
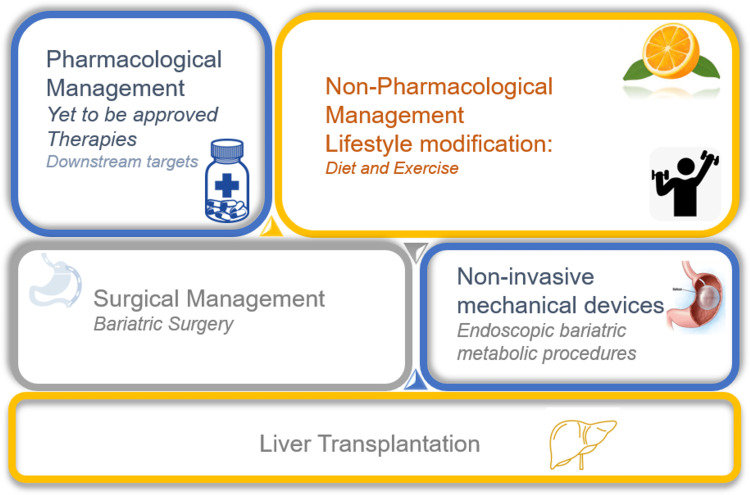
Management of NASH NASH-non-alcoholic steatohepatitis

Based on the currently available evidence combined diet/exercise strategies are synergistic and therefore more effective in reducing elevated liver enzyme levels, hepatic steatosis and improving histology than either modality alone, hence a holistic lifestyle change would be more beneficial than either intervention alone (Figure 3).

Pharmacological and surgical interventions: Weight loss and its maintenance are challenging consequently a larger patient pool may need alternate methods to restrict excessive calories [115]. These include patients unable to achieve >5% loss of total body weight with lifestyle interventions or unable to sustain, or who have a BMI ≥27 kg/m^2^ along with ≥1 metabolic comorbidity, or those with a BMI >30 kg/m^2^ with comorbidities. Several pharmacological agents which can achieve weight loss through different mechanisms are recommended in these patient groups. However, among the plethora of approved medications only one glucagon-like peptide-1 (GLP-1) agonist, has been found to improve liver histology in NASH patients in a phase II study [116]. Sodium-glucose cotransporter-2 (SGLT2) inhibitors have also been shown to be effective for weight loss and reduced hepatic fat content and may thus be beneficial in NASH [117]. However, the need for chronic use, monitoring and associated adverse events and sparse clinical evidence for improvement of NASH parameters limit the utility of these medications. Therefore, preference should be given to lifestyle interventions.

Bariatric surgery has also been evaluated for weight loss and extended benefits in patients with NASH. The largest prospective study demonstrated a likely deterioration of fibrosis, with an increase in the severity of fibrosis in ~20% of patients during a one-year follow-up period [118]. However, a recent study in severely obese patients with biopsy-proven NASH found the resolution of NASH in 84% of patients with progressive and sustained reduction of fibrosis that began during the first year and was sustained through five years [119]. Yet, the potential benefits must be weighed against the risks of bariatric surgery, particularly in patients with advanced disease conditions [120]. Further up to 20% of patients have normal BMI, in these patients with less severe obesity and related complications, including T2DM endoscopic bariatric metabolic therapies (EBMT) are rapidly emerging as less invasive and costly therapies [121]. The most studied include a variety of intragastric balloons (IGB), endoscopic sleeve gastroplasty (ESG) and duodenal mucosal resurfacing (DMR) [120]. IGBs and ESG have been shown to achieve adequate and sustained weight loss required for NASH improvement without significant adverse events [122]. These EMBTs can bridge the gap between lifestyle interventions having high safety but limited efficacy, while bariatric surgery has high efficacy with a poor safety profile [123]. The last resort is liver transplantation indicated by practice guidelines for patients with NASH and ESLD or HCC only. However, a study has shown that within a month of LT, 40% of patients with NASH were identified to be at risk of developing renal dysfunction implying serious safety issues in this patient population. An alternative investigated in patients predominantly suffering from NASH (>50% NASH cirrhosis) includes sleeve gastrectomy during LT which maintained greater weight loss and had fewer components of the metabolic syndrome [124]. Thus, a reduction in risk factors for post-LT metabolic syndrome may confer a significant survival benefit.

Current Drug Therapies Targeting Downstream and Upstream Pathways

Among the older drug classes, studies with pioglitazone demonstrated improved hepatic steatosis, ballooning necrosis, inflammation and fibrosis in steatohepatitis patients with or without prediabetes or T2DM [125-128]. However, these beneficial effects of pioglitazone are offset by an increased risk of weight gain, oedema, the development of bladder cancer and a decrease in bone mineral density [129]. Few clinical trials demonstrated significant reductions in steatosis, lobular inflammation and fibrosis in patients treated with vitamin E (400 IU) [130-132]. However, it was associated with an increased risk of all-cause mortality linked to hemorrhagic stroke and prostate cancer [131-133].

With a better understanding of the NASH pathogenesis and identification of target pathways, various drug classes are being evaluated for their efficacy. Majorly the drug classes target either metabolic pathways, fibrosis or oxidative stress. The drug classes acting on the metabolic pathways have demonstrated some clinical benefit due to the inhibition of de novo lipogenesis, improved insulin sensitivity, the rectification of the link between de novo lipogenesis and bile acid metabolism, increased β-oxidation of fatty acids in the mitochondria, and modulation of the uptake of fatty acids in the liver via thyroid hormone receptors [134-136]. The drugs are currently in phase IIb and phase III trials and their detailed description has been depicted in Table 7 [133,134,137-156]. The clinical outcomes of most of the remaining investigational drug classes have been disappointing.

**Table 7:** Investigational Drugs for The Treatment of NASH ACC-Acetyl-coenzyme A carboxylase, ALT-Alanine transaminase, ARREST-Aramchol for the REsolution of Steatohepatitis, ARRIVE-aramchol for HIV-associated NAFLD and lipodystrophy (ARRIVE) trial, ASK-1-apoptosis signal-regulating kinase 1, AST-Aspartate transaminase, CAP-controlled attenuation parameter, CCR-chemokine receptor, CCR2/CCR5-dual chemokine receptor, DGAT2-diacylglycerol acyltransferase 2, DNL-de-novo Lipogenesis, DCGI-Drug Controller General of India, ENCORE-Emricasan, an Oral Caspase Inhibitor, in Subjects With NASH Cirrhosis and Severe Portal Hypertension, FGF-fibroblast growth factor, FLIGHT-FXR-Study of Safety and Efficacy of Tropifexor (LJN452) in Patients With Non-alcoholic Steatohepatitis (NASH), farsenoid-x-receptor, FLINT-Farnesoid X nuclear receptor ligand obeticholic acid for non-cirrhotic, non-alcoholic steatohepatitis, GIP-glucose-dependent insulinotropic polypeptide, GLP-glucagon-like peptide, JNK-c-Jun amino-terminal kinase, LSM-Liver stiffness measurement, MPC-mitochondrial pyruvate carrier, NATIVE-NASH to Assess IVA337, SCD1-Stearoyl-coenzyme A desaturase 1, THR-thyroid hormone receptor, PPAR-peroxisome proliferator activated receptor, RESOLVE-IT-Study to Evaluate the Efficacy and Safety of Elafibranor Versus Placebo in Patients With Nonalcoholic Steatohepatitis, STELLAR-Safety and Efficacy of Selonsertib in Adults With Nonalcoholic Steatohepatitis and Bridging (F3) Fibrosis TANDEM-Study of Safety, Tolerability, and Efficacy of a Combination Treatment of LJN452 and CVC in Adult Patients With NASH and Liver Fibrosis, THR-hyroid hormone receptor, USFDA-United States Food and Drug Administration

Name of the Pathway	Mechanism	Drug(s)	Phase	Clinical Outcome
Liver-targeted ACC inhibitor and DGAT2	Inhibition ACC is the first committed enzyme in the hepatic DNL pathway. DGAT2 is highly expressed in the liver and adipose tissue and catalyses the terminal step of DNL, specifically the esterification of a fatty acid with diacylglycerol to form triglyceride. Independent inhibition of each of these steps has been shown to reduce hepatic steatosis	Ervogastat (DGAT2i) + Clesacostat (ACCi)	II	Metabolic Interventions to Resolve NASH with fibrosis-68-week study-results awaited
FXR agonist	FXR nuclear receptors are expressed in the liver and intestines. Activation reduces bile acid synthesis and uptake of bile acids in the ileum by downregulating the sodium-dependent bile acid transporter. Regulates cholesterol lipoprotein and bile acid metabolism to modulate immuno-inflammatory and fibrogenic responses	Obeticholic acid	III	FLINT: Improved the histological features No significant resolution of NASH REGENERATE: improvement in fibrosis by 23%
		Tropifexor	IIb	FLIGHTFXR: Recruitment TANDEM: Recruitment
PPAR-α/δ agonist:	Regulates lipid and insulin metabolism	Seladelpar	IIb	Phase 3, discontinued
		Lanifibranor	IIb	Awaiting Phase 3 NATIVE: Resolution of NASH and Fibrosis without worsening of either
		Elafibranor	III	GOLDEN: NASH was resolved without the worsening of fibrosis in 19% RESOLVE-IT: No histological benefit, discontinued
		Saroglitazar	Approved by DCGI, India (not USFDA)	significant improvement in transaminases, LSM, CAP, glycemic control, and lipid parameters
THR-ß agonist:	THR β is highly expressed in hepatocytes and is responsible for regulating the metabolic pathways in the liver that are frequently impaired in NAFLD and NASH	Resmetirom]	III	Potential to be approved Relative decrease in liver fat Significant improvement in steatohepatitis Significant reductions in ALT and AST levels, atherogenic lipids, lipo-protein(a), markers of inflammation and fibrosis as well as improvement in NASH on liver biopsies
FGF analogues	Regulates bile acid synthesis, glucose homoeostasis and energy homoeostasis	Pegbelfermin	IIb	Reduction in liver fat content with an acceptable safety profile
		NGM282	IIb	
MPC inhibitor	Insulin sensitizer that has been shown in initial studies to increase lipid oxidation and reduce de novo lipid synthesis and gluconeogenesis in the liver	MSDC-0602K	IIb	Phase 2/3 No Significant effects
GLP-1-GIP co-agonist	Improves glucose disposal	Tirzepatide	III	Improves glucose disposal and also reduces nausea associated with GLP-1 activity Significant weight loss in obese individuals

## Conclusions

Given the rising incidence, associated serious morbidities, and growing need for liver transplantation, it is vital to develop more accurate diagnostic methods to screen, recognize and diagnose NASH. Though liver biopsy is the only validated method of confirmation of diagnosis there has been significant progress in the development of alternative biomarkers and imaging modalities. These newer methods have provided an improved understanding of the pathological changes in the structure and function of the liver in recent years. Weight loss through lifestyle intervention still remains the cornerstone of NASH therapy, maintaining adherence which is truly challenging and subjective. Though bariatric surgery shows significant advantages in terms of the decline of fibrosis, patients with advanced disease are not appropriate candidates for it. Further lean NASH patients have been shown to develop more severe disease than obese patients, for whom pharmacotherapy is the only alternative. A few existing treatment alternatives include the use of therapies like SGLT2i or antioxidants and vitamin E which have shown some benefits in patients with NASH. However, there is a need for larger randomized trials with these drugs specifically in patients with NASH demonstrating histological benefits, attenuating progression to fibrosis and HCC. With an enhanced understanding of the pathogenesis of NASH, several newer drug classes targeting different sites are being investigated. Most agents are in phase 2 or 3 of their developmental phase. Currently, though results with certain classes like dual chemokine receptor (CCR2/CCR5) inhibitor, stearoyl-coenzyme A desaturase (SCD1) inhibitor, apoptosis signal-regulating kinase1 (ASK1) inhibitor III and caspase inhibitor have been disappointing few have shown encouraging results. These include glucagon-like peptide-1-glucose-dependent insulinotropic polypeptide (GLP-1-GIP) co-agonist, fibroblast growth factor (FGF analogs), thyroid hormone receptor-ß (THR-ß) agonist, few peroxisome proliferator-activated receptor-α/δ (PPAR-α/δ) agonist, farnesoid-x-receptor (FXR) agonist and diacylglycerol acyltransferase 2/Acetyl-coenzyme A carboxylase (DGAT2i/ACCi). These agents have shown a reduction in hepatic fat content, attenuation of fibrosis and a good tolerability profile in phase II studies. The results of a larger phase 3 study with these agents will end the long wait for an effective and well-tolerated universally approved drug class for the treatment of NASH.

NAFLD and NASH are driven by the obesity and diabetes epidemic, poor lifestyle, compounded by dysbiosis and influenced by genetic factors in others. Ongoing research has improved our comprehension of disease pathogenesis but means to diagnose and treat this progressive condition are still limited. These aspects should become a new priority given the poor outcomes with associated hepatic and extrahepatic events. Though not completely successful, the developments in the management of NASH have been fairly encouraging. Further well-designed long-term prospective studies should be undertaken to generate evidence with definitive results.
